# The effects of tobacco control policies on global smoking prevalence

**DOI:** 10.1038/s41591-020-01210-8

**Published:** 2021-01-21

**Authors:** Luisa S. Flor, Marissa B. Reitsma, Vinay Gupta, Marie Ng, Emmanuela Gakidou

**Affiliations:** 1 Institute for Health Metrics and Evaluation, Department of Health Metrics Sciences, University of Washington, Seattle, WA USA; 2 IBM Watson Health, San Jose, CA USA

**Keywords:** Risk factors, Policy

## Abstract

Substantial global effort has been devoted to curtailing the tobacco epidemic over the past two decades, especially after the adoption of the Framework Convention on Tobacco Control^1^ by the World Health Organization in 2003. In 2015, in recognition of the burden resulting from tobacco use, strengthened tobacco control was included as a global development target in the 2030 Agenda for Sustainable Development^2^. Here we show that comprehensive tobacco control policies—including smoking bans, health warnings, advertising bans and tobacco taxes—are effective in reducing smoking prevalence; amplified positive effects are seen when these policies are implemented simultaneously within a given country. We find that if all 155 countries included in our counterfactual analysis had adopted smoking bans, health warnings and advertising bans at the strictest level and raised cigarette prices to at least 7.73 international dollars in 2009, there would have been about 100 million fewer smokers in the world in 2017. These findings highlight the urgent need for countries to move toward an accelerated implementation of a set of strong tobacco control practices, thus curbing the burden of smoking-attributable diseases and deaths.

## Abstract (web summary)

Analysis of global smoking prevalence trends from 2009 to 2017 demonstrates that, when implemented, national-level tobacco control policies are highly effective; however, considerable gaps remain in the universal adoption of anti-tobacco interventions.

## Main

Decades after its ill effects on human health were first documented, tobacco smoking remains one of the major global drivers of premature death and disability. In 2017, smoking was responsible for 7.1 (95% uncertainty interval (UI), 6.8–7.4) million deaths worldwide and 7.3% (95% UI, 6.8%–7.8%) of total disability-adjusted life years^3^. In addition to the health impacts, economic harms resulting from lost productivity and increased healthcare expenditures are also well-documented negative effects of tobacco use^4,5^. These consequences highlight the importance of strengthening tobacco control, a critical and timely step as countries work toward the 2030 Sustainable Development Goals^2^.

In 2003, the World Health Organization (WHO) led the development of the Framework Convention on Tobacco Control (FCTC), the first global health treaty intended to bolster tobacco use curtailment efforts among signatory member states^1^. Later, in 2008, to assist the implementation of tobacco control policies by countries, the WHO introduced the MPOWER package, an acronym representing six evidence-based control measures (Table 1) (ref. ^6^). While accelerated adoption of some of these demand reduction policies was observed among FCTC parties in the past decade^7^, many challenges remain to further decrease population-level tobacco use. Given the differing stages of the tobacco epidemic and tobacco control across countries, consolidating the evidence base on the effectiveness of policies in reducing smoking is necessary as countries plan on how to do better. In this study, we evaluated the association between varying levels of tobacco control measures and age- and sex-specific smoking prevalence using data from 175 countries and highlighted missed opportunities to decrease smoking rates by predicting the global smoking prevalence under alternative unrealized policy scenarios.

**Table 1:** The WHO MPOWER policy package

MPOWER component	Definition
**M**	Monitor tobacco use and prevention policies
**P**	Protect people from tobacco smoke
**O**	Offer help to quit tobacco use
**W**	Warn about the dangers of tobacco
**E**	Enforce bans on tobacco advertising, promotion and sponsorship
**R**	Raise taxes on tobacco

MPOWER is a policy package intended to assist in the country-level implementation of cost-effective interventions to prevent and reduce tobacco use, as ratified by the WHO FCTC. Other than demand reduction measures, MPOWER encourages countries to develop surveillance systems to monitor the tobacco epidemic. Each of the MPOWER components reflects one or more provisions described in the FCTC.

**Extended Data Fig. 1:**
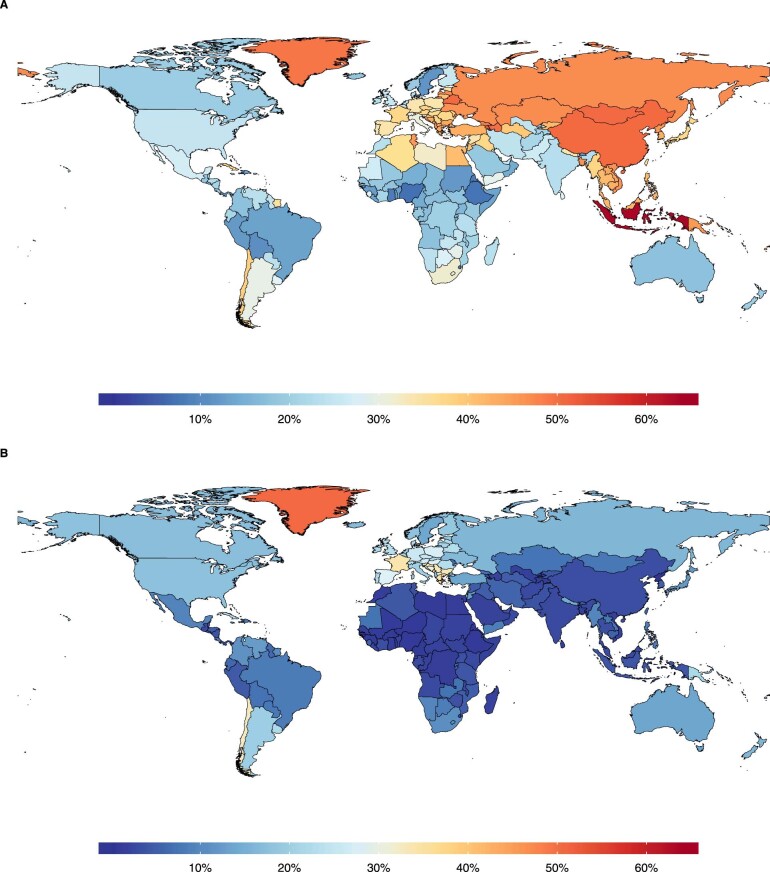
Prevalence of current smoking for men (a) and women (b) aged 15 years and older (age-standardized) in 2017. Age-standardized smoking prevalence (%) estimates from the 2017 Global Burden of Disease Study for men (a) and women (b) aged 15 years and older for 195 countries. Smoking is defined as current use of any type of smoked tobacco product. Details on the estimation process can be found in the Methods section and elsewhere^3^. Source data

**Extended Data Fig. 2:**
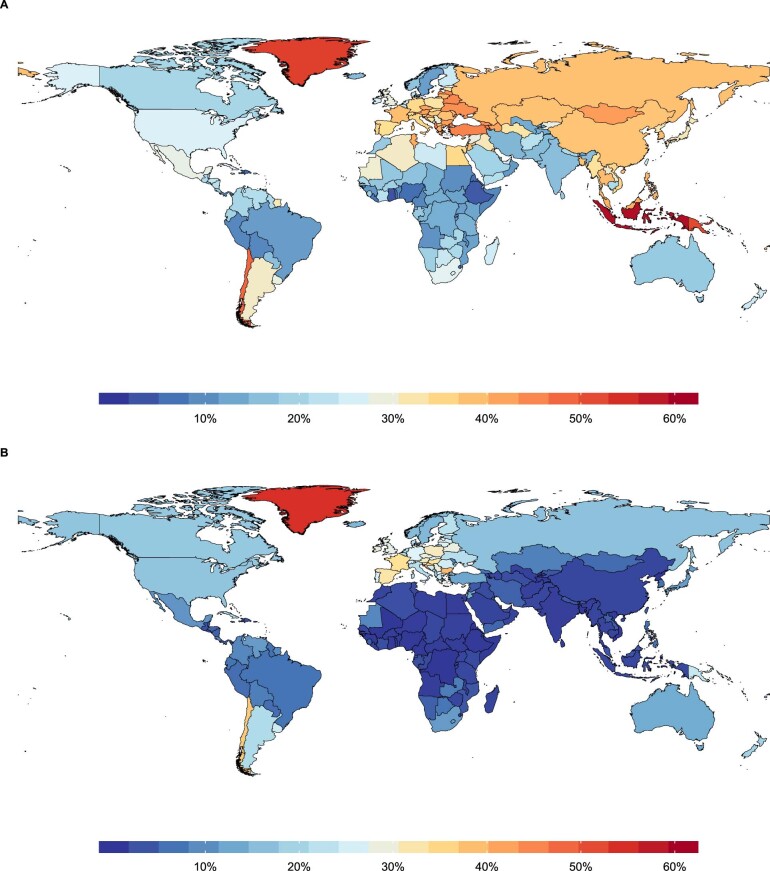
Prevalence of current smoking for men (a) and women (b) aged 15 to 29 years old (age-standardized) in 2017. Age-standardized smoking prevalence (%) estimates from the 2017 Global Burden of Disease Study for men (a) and women (b) aged 15–29 years old for 195 countries. Smoking is defined as current use of any type of smoked tobacco product. Details on the estimation process can be found in the Methods section and elsewhere^3^. Source data

**Extended Data Fig. 3:**
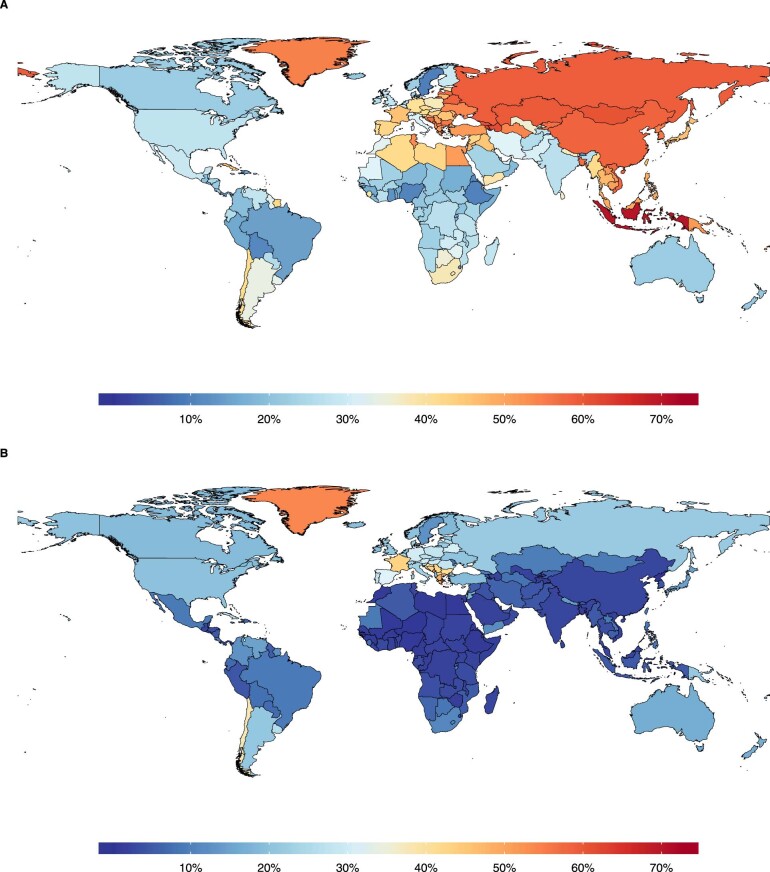
Prevalence of current smoking for men (a) and women (b) aged 30 to 49 years old (age-standardized) in 2017. Age-standardized smoking prevalence (%) estimates from the 2017 Global Burden of Disease Study for men (a) and women (b) aged 30–49 years old for 195 countries. Smoking is defined as current use of any type of smoked tobacco product. Details on the estimation process can be found in the Methods section and elsewhere^3^. Source data

**Extended Data Fig. 4:**
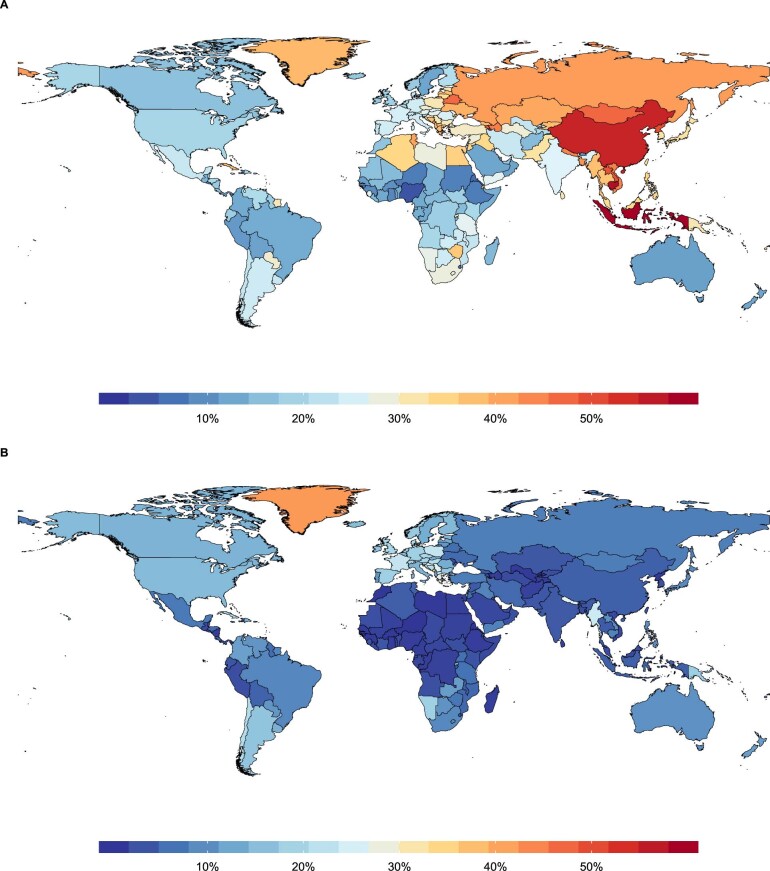
Prevalence of current smoking for men (a) and women (b) aged 50 years and older (age-standardized) in 2017. Age-standardized smoking prevalence (%) estimates from the 2017 Global Burden of Disease Study for men (a) and women (b) aged 50 years and older for 195 countries. Smoking is defined as current use of any type of smoked tobacco product. Details on the estimation process can be found in the Methods section and elsewhere^3^. Source data

Despite the enhanced global commitment to control tobacco use, the pace of progress in reducing smoking prevalence has been heterogeneous across geographies, development status, sex and age^8^; in 2017, there were still 1.1 billion smokers across the 195 countries and territories assessed by the Global Burden of Diseases, Injuries, and Risk Factors Study. Global smoking prevalence in 2017 among men and women aged 15 and older, 15–29 years, 30–49 years and 50 years and older are shown in Extended Data Figs. 1, 2, 3 and 4, respectively. We found that, between 2009 and 2017, current smoking prevalence declined by 7.7% for men (36.3% (95% UI, 35.9–36.6%) to 33.5% (95% UI, 32.9–34.1%)) and by 15.2% for women globally (7.9% (95% UI, 7.8–8.1%) to 6.7% (95% UI, 6.5–6.9%)). The highest relative decreases were observed among men and women aged 15–29 years, at 10% and 20%, respectively. Conversely, prevalence decreased less intensively for those aged over 50, at 2% for men and 9.5% for women. While some countries have shown an important reduction in smoking prevalence between 2009 and 2017, such as Brazil, suggesting sustained progress in tobacco control, a handful of countries and territories have shown considerable increases in smoking rates among men (for example, Albania) and women (for example, Portugal) over this time period.

**Fig. 1:**
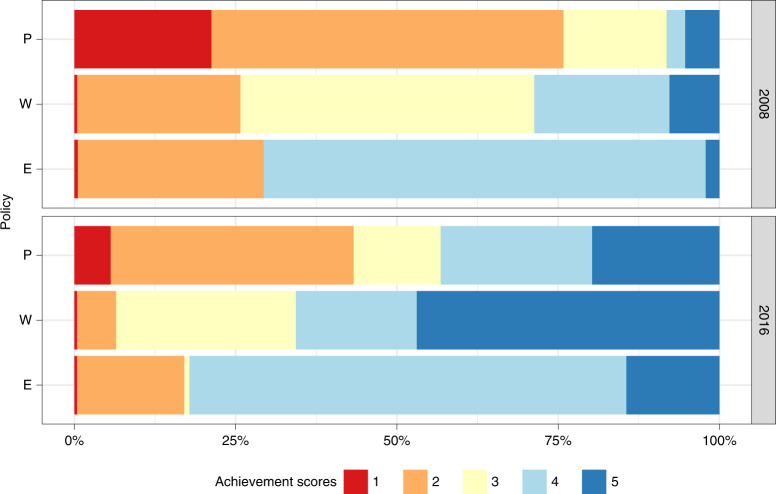
Level of coverage of the population aged 15 years and older by comprehensive smoke-free, health warning and advertising policies in 2008 and in 2016. To assess national-level legislation quality, the WHO attributes a score to each MPOWER component that ranges from 1 to 5 for smoke-free (P), health warning (W) and advertising (E) policies. A score of 1 represents no known data or no recent data, while scores 2–5 characterize the overall strength of each policy, from 2 representing the lowest level of achievement (weakest policy), to 5 representing the highest level of achievement (strongest policy). Source data

In an effort to counteract the harmful lifelong consequences of smoking, countries have, overall, implemented stronger demand reduction measures after the FCTC ratification. To assess national-level legislation quality, the WHO attributes a score to each of the MPOWER measures that ranges from 1 to 4 for the monitoring component (M) and 1–5 for the other components. A score of 1 represents no known data, while scores 2–5 characterize the overall strength of each measure, from the lowest level of achievement (weakest policy) to the highest level of achievement (strongest policy)^6^. Between 2008 and 2016, although very little progress was made in treatment provision (O)^7,9^, the share of the total population covered by best practice (score = 5) P, W and E measures increased (Fig. 1). Notably, however, a massive portion of the global population is still not covered by comprehensive laws. As an example, less than 15% of the global population is protected by strongly regulated tobacco advertising (E) and the number of people (2.1 billion) living in countries where none or very limited smoke-free policies (P) are in place (score = 2) is still nearly twice as high as the population (1.1 billion) living in locations with national bans on smoking in all public places (score = 5).

In terms of fiscal policies (R), the population-weighted average price, adjusted for inflation, of a pack of cigarettes across 175 countries with available data increased from I$3.10 (where I$ represents international dollars) in 2008 to I$5.38 in 2016. However, from an economic perspective, for prices to affect purchasing decisions, they need to be evaluated relative to income. The relative income price (RIP) of cigarettes is a measure of affordability that reflects, in this study, what proportion of the country-specific per capita gross domestic product (GDP) is needed to purchase half a pack of cigarettes a day for a year. Over time, cigarettes have become less affordable (RIP 2016 > RIP 2008) in about 75% of the analyzed countries, with relatively more affordable cigarettes concentrated across high-income countries.

Our adjusted analysis indicates that greater levels of achievement on key measures across the P, W and E policy categories and higher RIP values were significantly associated with reduced smoking prevalence from 2009 to 2017 (Table 2). Among men aged 15 and older, each 1-unit increment in achievement scores for smoking bans (P) was independently associated with a 1.1% (95% UI, −1.7 to −0.5, *P* < 0.0001) decrease in smoking prevalence. Similarly, an increase of 1 point in W and E scores was associated with a decrease in prevalence of 2.1% (95% UI, −2.7 to −1.6, *P* < 0.0001) and 1.9% (95% UI, −2.6 to −1.1, *P* < 0.0001), respectively. Furthermore, a 10 percentage point increase in RIP was associated with a 9% (95% UI, −12.6 to −5.0, *P* < 0.0001) decrease in overall smoking prevalence. Results were similar for men from other age ranges.

**Table 2:** Percentage changes in current smoking prevalence based on fixed effect coefficients from adjusted mixed effect linear regression models, by policy component, sex and age group

Age and policy component	Men			Women			Both sexes		
	Relative change	95% UI	*P*	Relative change	95% UI	*P*	Relative change	95% UI	*P*
≥15									
RIP	−0.9	−1.3 to −0.5	<0.001	−0.6	−1.0 to −0.2	0.014	−0.7	−1.1 to −0.4	0.001
P	−1.1	−1.7 to −0.5	<0.001	−0.9	−1.7 to 0.1	0.065	−1.0	−1.6 to −0.5	0.001
W	−2.1	−2.7 to −1.6	<0.001	−3.6	−4.5 to −2.9	<0.001	−2.1	−2.6 to −1.5	<0.001
E	−1.9	−2.6 to −1.1	<0.001	−1.9	−2.9 to −1.8	0.002	−1.8	−2.5 to −1.8	<0.001
15–29									
RIP	−1.1	−1.6 to −0.6	<0.001	−0.8	−1.5 to −0.1	0.016	−1.1	−1.6 to −0.6	0.001
P	−1.0	−1.7 to −0.3	0.003	−0.8	−1.9 to 0.4	0.177	−0.9	−1.6 to −0.3	0.008
W	−2.1	−2.7 to −1.4	<0.001	−4.0	−5.0 to −3.0	<0.001	−2.1	−2.8 to −1.4	<0.001
E	−2.2	−3.1 to −2.2	<0.001	−2.5	−4.0 to −2.5	<0.001	−2.1	−2.9 to −1.1	<0.001
30–49									
RIP	−1.1	−1.6 to −0.6	<0.001	−0.6	−1.1 to −0.1	0.016	−0.9	−1.3 to −0.4	0.001
P	−1.1	−1.8 to −0.5	<0.001	−1.3	−2.3 to −0.2	0.016	−1.2	−1.8 to −0.6	0.001
W	−2.4	−3.0 to −1.8	<0.001	−3.5	−4.5 to −2.6	<0.001	−2.2	−2.8 to −1.5	<0.001
E	−1.7	−2.5 to −1.7	<0.001	−1.7	−3.1 to −1.7	0.010	−1.6	−2.5 to −1.6	0.001
≥50									
RIP	−0.6	−0.9 to −0.3	<0.001	−0.2	−0.3 to 0.0	0.119	−0.3	−0.6 to −0.1	0.011
P	−1.2	−1.8 to −0.6	<0.001	−0.1	−1.0 to 0.8	0.797	−1.0	−1.5 to −0.4	0.001
W	−1.9	−2.4 to −1.3	<0.001	−2.9	−3.7 to −2.1	<0.001	−1.7	−2.2 to −1.2	<0.001
E	−1.9	−2.7 to −1.9	<0.001	−2.0	−3.0 to −2.0	<0.001	−1.9	−2.6 to −1.9	<0.001

The models examined the adjusted association between smoke-free (P), health warning (W) and advertising (E) achievement scores, as well as cigarette’s affordability (RIP) and current smoking prevalence, from 2009 to 2017, across 175 countries (*n* = 823 country-years). Linear mixed models were fitted by maximum likelihood and *t*-tests used Satterthwaite approximations to degrees of freedom. *P* values were considered statistically significant if <0.05.

Among women, the magnitude of effect of different policy indicators varied across age groups. For those aged over 15, each 1-point increment in W and E scores was independently associated with an average reduction in prevalence of 3.6% (95% UI, −4.5 to −2.9, *P* < 0.0001) and 1.9% (95% UI, −2.9 to −1.8, *P* = 0.002), respectively, and these findings were similar across age groups. Smoking ban (P) scores were not associated with reduced prevalence among women aged 15–29 years or over 50 years. However, a 1-unit increase in *P* scores was associated with a 1.3% (95% UI, −2.3 to −0.2, *P* = 0.016) decline in prevalence among women aged 30–49 years. Lastly, while a 10 percentage point increase in RIP lowered women smoking prevalence by 6% overall (95% UI, −10.0 to −2.0, *P*= 0.014), this finding was not statistically significant when examining reductions in prevalence among those aged 50 and older (Table 2).

If tobacco control had remained at the level it was in 2008 for all 155 countries (with non-missing policy indicators for both 2008 and 2016; Methods) included in the counterfactual analysis, we estimate that smoking prevalence would have been even higher than the observed 2017 rates, with 23 million more male smokers and 8 million more female smokers (age ≥ 15) worldwide (Table 3). Out of the counterfactual scenarios explored, the greatest progress in reducing smoking prevalence would have been observed if a combination of higher prices—resulting in reduced affordability levels—and strictest P, W and E laws had been implemented by all countries, leading to lower smoking rates among men and women from all age groups and approximately 100 million fewer smokers across all countries (Table 3). Under this policy scenario, the greatest relative decrease in prevalence would have been seen among those aged 15–29 for both sexes, resulting in 26.6 and 6.5 million fewer young male and female smokers worldwide in 2017, respectively.

**Table 3:** Observed and simulated reduction in global smoking prevalence and in the total number of smokers under four counterfactual scenarios, by sex and age group

Age and sex	Observed—GBD 2017		Scenario 1		Scenario 2		Scenario 3		Scenario 4	
	2017 prevalence (95% UI)	Number of smokers (in millions)	Relative % change in prevalence	Change in the number of smokers (in millions)	Relative % change in prevalence	Change in the number of smokers (in millions)	Relative % change in prevalence	Change in the number of smokers (in millions)	Relative % change in prevalence	Change in the number of smokers (in millions)
Men										
≥15	37.1 (36.4–37.8)	768.1	+3%	+22.7	−5.9%	−45.5	−4.9%	−37.9	−10.6%	−81.7
15–29	29.8 (28.6–30.9)	192.3	+3.5%	+6.7	−9.2%	−17.4	−5.2%	−9.9	−14%	−26.6
30–49	44.1 (42.7–45.4)	336.9	+2.9%	+9.7	−6.3%	−20.7	−4.6%	−15.3	−10.6%	−35.4
≥50	37 (35.8–38.3)	238.9	+2.6%	+6.3	−3.2%	−7.4	−5.3%	−12.7	−8.4%	−19.7
Women										
≥15	7.4 (7.1–7.6)	156	+4.9%	+7.6	−5%	−7.8	−7.3%	−11.4	−12%	−18.7
15–29	6.1 (5.7–6.5)	38.7	+6.1%	+2.4	−8.4%	−3.5	−8.1%	−3.3	−15.9%	−6.5
30–49	8.1 (7.7–8.5)	61.4	+4.9%	+3.2	−5.1%	−3.4	−7.8%	−4.9	−12.5%	−8.1
≥50	7.9 (7.4–8.5)	55.9	+3.4%	+2	−1%	−0.9	−5.5%	−3.2	−6.5%	−4.1

Prevalence and number of smokers across 155 countries included in the counterfactual analysis (non-missing policy/price indicators for both 2008 and 2016). Alternative policy scenarios are: (1) if achievement scores and cigarette’s affordability (RIP) remained at the level they were at in 2008; (2) if the price of a cigarette pack was I$7.73 or higher, a price that represents the 90th percentile of observed prices across all 175 countries and years; (3) if all countries had implemented each of the smoke-free (P), health warning (W) and advertising (E) policies at the highest level (score = 5); and (4) if countries had implemented both higher cigarette prices and P, W and E policies at the highest level.

Our findings reaffirm that a wide spectrum of tobacco demand reduction policies has been effective in reducing smoking prevalence globally; however, it also indicates that even though much progress has been achieved, there is considerable room for improvement and efforts need to be strengthened and accelerated to achieve additional gains in global health. A growing body of research points to the effectiveness of tobacco control measures^10–12^; however, this study covers the largest number of countries and years so far and reveals that the observed impact has varied by type of control policy and across sexes and age groups. In high-income countries, stronger tobacco control efforts are also associated with higher cessation ratios (that is, the ratio of former smokers divided by the number of ever-smokers (current and former smokers)) and decreases in cigarette consumption^13,14^.

Specifically, our results suggest that men are, in general, more responsive to tobacco control interventions compared to women. Notably, with prevalence rates for women being considerably low in many locations, variations over time are more difficult to detect; thus, attributing causes to changes in outcome can be challenging. Yet, there is already evidence that certain elements of tobacco control policies that play a role in reducing overall smoking can have limited impact among girls and women, particularly those of low socioeconomic status^15^. Possible explanations include the different value judgments attached to smoking among women with respect to maintaining social relationships, improving body image and hastening weight control^16^.

Tax and price increases are recognized as the most impactful tobacco control policy among the suite of options under the MPOWER framework^10,14,17^, particularly among adolescents and young adults^18^. Previous work has also demonstrated that women are less sensitive than men to cigarette tax increases in the USA^19^. Irrespective of these demographic differences, effective tax policy is underutilized and only six countries—Argentina, Chile, Cuba, Egypt, Palau and San Marino—had adopted cigarette taxes that corresponded to the WHO-prescribed level of 70% of the price of a full pack by 2017 (ref. ^20^). Cigarettes also remain highly affordable in many countries, particularly among high-income nations, an indication that affordability-based prescriptions to countries, instead of isolated taxes and prices reforms, are possibly more useful as a tobacco control target. In addition, banning sales of single cigarettes, restricting legal cross-border shopping and fighting illicit trade are required so that countries can fully experience the positive effect of strengthened fiscal policies.

Smoke-free policies, which restrict the opportunities to smoke and decrease the social acceptability of smoking^17^, also affect population groups differently. In general, women are less likely to smoke in public places, whereas men might be more frequently influenced by smoking bans in bars, restaurants, clubs and workplaces across the globe due to higher workforce participation rates^16^. In addition to leading to reduced overall smoking rates, as indicated in this study, implementing complete smoking bans (that is, all public places completely smoke-free) at a faster pace can also play an important role in minimizing the burden of smoking-attributable diseases and deaths among nonsmokers. In 2017 alone, 2.18% (95% UI, 1.8–2.7%) of all deaths were attributable to secondhand smoke globally, with the majority of the burden concentrated among women and children^21^.

Warning individuals about the harms of tobacco use increases knowledge about the health risks of smoking and promotes changes in smoking-related behaviors, while full advertising and promotion bans—implemented by less than 20% of countries in 2017 (ref. ^20^)—are associated with decreased tobacco consumption and smoking initiation rates, particularly among youth^17,22,23^. Large and rotating pictorial graphic warnings are the most effective in attracting smokers’ attention but are lacking in countries with high numbers of smokers, such as China and the USA^20^. Adding best practice health warnings to unbranded packages seems to be an effective way of informing about the negative effects of smoking while also eliminating the tobacco industry’s marketing efforts of using cigarette packages to make these products more appealing, especially for women and young people who are now the prime targets of tobacco companies^24,25^.

While it is clear that strong implementation and enforcement are crucial to accelerating progress in reducing smoking and its burden globally, our heterogeneous results by type of policy and demographics highlight the challenges of a one-size-fits-all approach in terms of tobacco control. The differences identified illustrate the need to consider the stages^26^ of the smoking epidemics among men and women and the state of tobacco control in each country to identify the most pressing needs and evaluate the way ahead. Smoking patterns are also influenced by economic, cultural and political determinants; thus, future efforts in assessing the effectiveness of tobacco control policies under these different circumstances are of value. As tobacco control measures have been more widely implemented, tobacco industry forces have expanded and threaten to delay or reverse global progress^27^. Therefore, closing loopholes through accelerated universal adoption of the comprehensive set of interventions included in MPOWER, guaranteeing that no one is left unprotected, is an urgent requirement as efforts toward achieving the Sustainable Development Goals by 2030 are intensified.

## Methods

This was an ecological time series analysis that aimed to estimate the effect of four key demand reduction measures on smoking rates across 175 countries. Country-year-specific achievement scores for P, W and E measures and an affordability metric measured by RIP—to capture the impact of fiscal policy (R)—were included as predictors in the model. Although the WHO also calls for monitoring (M) and tobacco cessation (O) interventions, these were not evaluated. Monitoring tobacco use is not considered a demand reduction measure, while very little progress has been made in treatment provision over the last decade^7,9^. Further information on research design is available in the Life Sciences Reporting Summary linked to this paper.

### Smoking outcome data

The dependent variable is represented by country-specific, age-standardized estimates of current tobacco smoking prevalence, defined as individuals who currently use any smoked tobacco product on a daily or occasional basis. Complete time series estimates of smoking prevalence from 2009 to 2017 for men and women aged 15–29, 30–49, 50 years and older and 15 years and older, were taken from the Global Burden of Disease (GBD) 2017 study.

The GBD is a scientific effort to quantify the comparative magnitude of health loss due to diseases, injuries and risk factors by age, sex and geography for specific points in time. While full details on the estimation process for smoking prevalence have been published elsewhere, we briefly describe the main analytical steps in this article^3^. First, 2,870 nationally representative surveys meeting the inclusion criteria were systematically identified and extracted. Since case definitions vary between surveys, for example, some surveys only ask about daily smoking as opposed to current smoking that includes both daily and occasional smokers, the extracted data were adjusted to the reference case definition using a linear regression fit on surveys reporting multiple case definitions. Next, for surveys with only tabulated data available, nonstandard age groups and data reported as both sexes combined were split using observed age and sex patterns. These preprocessing steps ensured that all data used in the modeling were comparable. Finally, spatiotemporal Gaussian process regression, a three-step modeling process used extensively in the GBD to estimate risk factor exposure, was used to estimate a complete time series for every country, age and sex. In the first step, estimates of tobacco consumption from supply-side data are incorporated to guide general levels and trends in prevalence estimates. In the second step, patterns observed in locations, age groups and years with smoking prevalence data are synthesized to improve the first-step estimates. This step is particularly important for countries and time periods with limited or no available prevalence data. The third step incorporates and quantifies uncertainty from sampling error, non-sampling error and the preprocessing data adjustments. For this analysis, the final age-specific estimates were age-standardized using the standard population based on GBD population estimates. Age standardization, while less important for the narrower age groups, ensured that the estimated effects of policies were not due to differences in population structure, either within or between countries.

Using GBD-modeled data is a strength of the study since nearly 3,000 surveys inform estimates and countries are not required to have complete survey coverage between 2009 and 2017 to be included in the analysis. Yet, it is important to note that these estimates have limitations. For example, in countries where a prevalence survey was not conducted after the enactment of a policy, modeled estimates may not reflect changes in prevalence resulting from that policy. Nonetheless, the prevalence estimates from the GBD used in this study are similar to those presented in the latest WHO report^28^, indicating the validity and consistency of said estimates.

### MPOWER data

Summary indicators of country-specific achievements for each MPOWER measure are released by the WHO every two years and date back to 2007. Data from different iterations of the WHO Report on the Global Tobacco Epidemic (2008^6^, 2009^29^, 2011^30^, 2013^31^, 2015^32^ and 2017^20^) were downloaded from the WHO Tobacco Free Initiative website (https://www.who.int/tobacco/about/en/). To assess the quality of national-level legislation, the WHO attributes a score to each MPOWER component that ranges from 1 to 4 for the monitoring (M) dimension and 1–5 for the other dimensions. A score of 1 represents no known data or no recent data, while scores 2–5 characterize the overall strength of each policy, from the lowest level of achievement (weakest policy) to the highest (strongest policy).

Specifically, smoke-free legislation (P) is assessed to determine whether smoke-free laws provide for a complete indoor smoke-free environment at all times in each of the respective places: healthcare facilities; educational facilities other than universities; universities; government facilities; indoor offices and workplaces not considered in any other category; restaurants or facilities that serve mostly food; cafes, pubs and bars or facilities that serve mostly beverages; and public transport. Achievement scores are then based on the number of places where indoor smoking is completely prohibited. Regarding health warning policies (W), the size of the warnings on both the front and back of the cigarette pack are averaged to calculate the percentage of the total pack surface area covered by the warning. This information is combined with seven best practice warning characteristics to construct policy scores for the W dimension. Finally, countries achievements in banning tobacco advertising, promotion and sponsorship (E) are assessed based on whether bans cover the following types of direct and indirect advertising: (1) direct: national television and radio; local magazines and newspapers; billboards and outdoor advertising; and point of sale (indoors); (2) indirect: free distribution of tobacco products in the mail or through other means; promotional discounts; nontobacco products identified with tobacco brand names; brand names of nontobacco products used or tobacco products; appearance of tobacco brands or products in television and/or films; and sponsorship.

P, W and E achievement scores, ranging from 2 to 5, were included as predictors into the model. The goal was to not only capture the effect of adopting policies at its highest levels but also assess the reduction in prevalence that could be achieved if countries moved into the expected direction in terms of implementing stronger measures over time. Additionally, having P, W and E scores separately, and not combined into a composite score, enabled us to capture the independent effect of different types of policies.

Although compliance is a critical factor in understanding policy effectiveness, the achievement scores incorporated in our main analysis reflect the adoption of legislation rather than degree of enforcement, representing a limitation of these indicators.

### Price data

Prices in I$ for a 20-cigarette pack of the most sold brand in each of the 175 countries were also sourced from the WHO Tobacco Free Initiative website for all available years (2008, 2010, 2012, 2014 and 2016). I$ standardize prices across countries and also adjust for inflation across time. This information was used to construct an affordability metric that captures the impact of cigarette prices on smoking prevalence, considering the income level of each country.

More specifically, the RIP, calculated as the percentage of per capita GDP required to purchase one half pack of cigarettes a day over the course of a year, was computed for each available country and year. Per capita GDP estimates were drawn from the Institute for Health Metrics and Evaluation; the estimation process is detailed elsewhere^33^.

Given that the price data used in the analysis refer to the most sold brand of cigarettes only, it does not reflect the full range of prices of different types of tobacco products available in each location. This might particularly affect our power in detecting a strong effect in countries where other forms of tobacco are more popular.

### Statistical analysis

**Extended Data Fig. 5:**
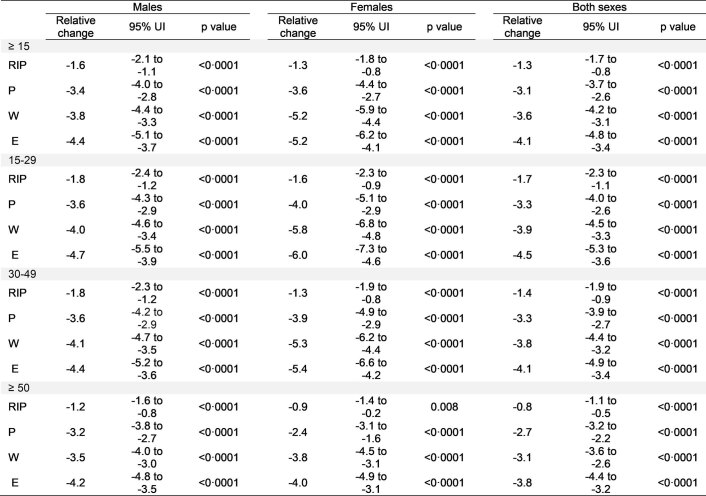
Percentage changes in current smoking prevalence based on fixed effect coefficients from bivariate mixed effect linear regression models, by policy component, sex and age group. Bivariate models examined the unadjusted association between smoke-free (P), health warnings (W), and advertising (E) achievement scores, and cigarette’s affordability (RIP) and current smoking prevalence, from 2009 to 2017, across 175 countries (n = 823 country-years). Linear mixed models were fit by maximum likelihood and t-tests used Satterthwaite approximations to degrees of freedom. P values were considered statistically significant if lower than 0.05.

Sex- and age-specific logit-transformed prevalence estimates from 2009 to 2017 were matched to one-year lagged achievement scores and RIP values using country and year identifiers^34^. The final sample consisted of 175 countries and was constrained to locations and years with non-missing indicators. A multiple linear mixed effects model fitted by restricted maximum likelihood was used to assess the independent effect of P, W and E scores and RIP values on the rates of current smoking. Specifically, a country random intercept and a country random slope on RIP were included to account for geographical heterogeneity and within-country correlation. The regression model takes the following general form:

$${\mathrm {logit}}\left( {y_{c,t}} \right) = \beta _0 + \beta _p{\mathrm {P}}_{c,t - 1} + \beta _w{\mathrm{W}}_{c,t - 1} + \beta _e{\mathrm{E}}_{c,t - 1} + \beta _r{\mathrm{R}}_{c,t - 1} + \alpha _c + \delta _c{\mathrm {R}}_{c,t - 1} + {\it{\epsilon }}_{c,t}$$

where *y*_*c,t*_ is the prevalence of current smoking in each country (*c*) and year (*t*), β_0_ is the intercept for the model and β_p_, β_w_, β_e_ and β_r_ are the fixed effects for each of the policy predictors. $\mathrm{P}_{c,\,t - 1},\,\mathrm{W}_{c,\,t - 1},\,\mathrm{E}_{c,\,t - 1}$ are the P, W and E scores and R_*c*,*t*−1_ is the RIP value for country *c* in year *t* − 1. Finally, α_*c*_ is the random intercept for country (*c*), while δ_*c*_ represent the random slope for the country (*c*) to which the RIP value (R_*t*−__1_) belongs. Variance inflation factor values were calculated for all the predictor parameters to check for multicollinearity; the values found were low (<2)^35^. Bivariate models were also run and are shown in Extended Data Fig. 5. The one-year lag introduced into the model may have led to an underestimation of effect sizes, particularly as many MPOWER policies require a greater period of time to be implemented effectively. However, due to the limited time range of our data (spanning eight years in total), introducing a longer lag period would have resulted in the loss of additional data points, thus further limiting our statistical power in detecting relevant associations between policies and smoking prevalence.

In addition to a joint model for smokers from both sexes, separate regressions were fitted for men and women and the four age groups (15–29, 30–49, ≥50 and ≥15 years old). To assess the validity of the mixed effects analyses, likelihood ratio tests comparing the models with random effects to the null models with only fixed effects were performed. Linear mixed models were fitted by maximum likelihood and *t*-tests used Satterthwaite approximations to degrees of freedom. *P* values were considered statistically significant if <0.05. All analyses were executed with RStudio v.1.1.383 using the lmer function in the R package lme4 v.1.1-21 (ref. ^36^).

A series of additional models to examine the impact of tobacco control policies were developed as part of this study. In each model, cigarette affordability (RIP) and a different set of policy metrics was used to capture the implementation, quality and compliance of tobacco control legislation. In models 1 and 2, we replaced the achievements scores by the proportion of P, W and E measures adopted by each country out of all possible measures reported by the WHO. In model 3, we used P and E (direct and indirect measures separately) compliance scores provided by the WHO to represent actual legislation implementation. Finally, an interaction term for compliance and achievement to capture the combined effect of legislation quality and performance was added to model 4. Results for men and women by age group for each of the additional models are presented in the Supplemental Information (Supplementary Tables 1–4).

The main model described in this study was chosen because it includes a larger number of country-year observations (*n* = 823) when compared to models including compliance scores and because it is more directly interpretable.

### Counterfactual analysis

To further explore and quantify the impact of tobacco control policies on current smoking prevalence, we simulated what smoking prevalence across all countries would have been achieved in 2017 under 4 alternative policy scenarios: (1) if achievement scores and RIP remained at the level they were at in 2008; (2) if all countries had implemented each of P, W and E component at the highest level (score = 5); (3) if the price of a cigarette pack was I$7.73 or higher, a price that represents the 90th percentile of observed prices across all countries and years; and (4) if countries had implemented the P, W and E components at the highest level and higher cigarette prices. To keep our results consistent across scenarios, we restricted our analysis to 155 countries with non-missing policy-related indicators for both 2008 and 2016.

Random effects were used in model fitting but not in this prediction. Simulated prevalence rates were calculated by multiplying the estimated marginal effect of each policy by the alternative values proposed in each of the counterfactual scenarios for each country-year. The global population-weighted average was computed for status quo and counterfactual scenarios using population data sourced from the Institute for Health Metrics and Evaluation. Using the predicted prevalence rates and population data, the additional reduction in the number of current smokers in 2017 was also computed. Since models were ran using age-standardized prevalence, the number of smokers was proportionally redistributed across age groups using the sex-specific numbers from the age group 15 and older as an envelope.

The UIs for predicted estimates were based on a computation of the results of each of the 1,000 draws (unbiased random samples) taken from the uncertainty distribution of each of the estimated coefficients; the lower bound of the 95% UI for the final quantity of interest is the 2.5 percentile of the distribution and the upper bound is the 97.5 percentile of the distribution.

### Reporting Summary

Further information on research design is available in the Nature Research Reporting Summary linked to this article.

## Online content

Any methods, additional references, Nature Research reporting summaries, source data, extended data, supplementary information, acknowledgements, peer review information; details of author contributions and competing interests; and statements of data and code availability are available at 10.1038/s41591-020-01210-8.

## Supplementary information

Supplementary Information Supplementary Tables 1–4: additional models results.

Reporting Summary

## Data Availability

The dataset generated and analyzed during the current study is publicly available at http://ghdx.healthdata.org/record/ihme-data/global-tobacco-control-and-smoking-prevalence-scenarios-2017 (10.6069/QAZ7-6505). The dataset contains all data necessary to interpret, replicate and build on the methods or findings reported in the article. Tobacco control policy data that support the findings of this study are released every two years as part of the WHO’s Global Report on Tobacco Control; these data are also directly accessible at https://www.who.int/tobacco/global_report/en/. Source data are provided with this paper.

## Source data

Source Data Fig. 1 Input data for Fig. 1 replication. Source Data Extended Data Fig. 1 Input data for Extended Data 1 replication. Source Data Extended Data Fig. 2 Input data for Extended Data 2 replication. Source Data Extended Data Fig. 3 Input data for Extended Data 3 replication. Source Data Extended Data Fig. 4 Input data for Extended Data 4 replication.

## Code availability

All code used for these analyses is available at http://ghdx.healthdata.org/record/ihme-data/global-tobacco-control-and-smoking-prevalence-scenarios-2017 and https://github.com/ihmeuw/team/tree/effects_tobacco_policies.
