# Supplementary material for: The effects of tobacco control policies on global smoking prevalence
Source: Nat Med. 2021 Jan 21;27(2):239–43. doi: 10.1038/s41591-020-01210-8 (PMC7884287; doi:10.1038/s41591-020-01210-8)
Supplement: Supplementary file 1 — Supplementary Tables 1–4: additional models results. [file 41591_2020_1210_MOESM1_ESM.pdf]

natureresearch

https://doi.org/10.1038/s41591-020-01210-8

Supplementary information

The effects of tobacco control policies on global smoking prevalence

In the format provided by the authors and unedited

Supplementary Table 1. Percent changes in current smoking prevalence based on fixed effect coefficients from adjusted mixed effect linear regression models (proportion adopted models), by policy component, sex and age group. Models examined the adjusted association between the proportion of smoke-free (%P), health warnings (%W), and advertising (%E) measures adopted and cigarette's affordability (RIP) and current smoking prevalence, from 2009 to 2017, across 175 countries (n=844). Linear mixed models were fit by maximum likelihood and t-tests used Satterthwaite approximations to degrees of freedom. P values were considered statistically significant if lower than 0.05.

	Men			Women		
	Relative change	95% UI	p value	Relative change	95% UI	p value
≥ 15						
RIP	-1.2	-1.5 to -0.7	<0.001	-0.8	-1.3 to -0.4	0.002
%P	-3.3	-5.8 to -1	0.007	-2.8	-6.6 to 0.8	0.128
%W	-11.6	-15.5 to -7.6	<0.001	-20.2	-25.9 to -14.3	<0.001
%E	-6.6	-9.5 to -6.6	<0.001	-5.7	-10.2 to -5.7	0.015
15-29						
RIP	-1.4	-1.9 to -0.9	<0.001	-1.1	-1.8 to -0.5	0.002
%P	-3.3	-6.2 to -0.6	0.018	-2.6	-7.5 to 1.9	0.266
%W	-10.2	-14.4 to -5.7	<0.001	-22.5	-30.1 to -14.7	<0.001
%E	-7.6	-11.1 to -7.6	<0.001	-7.6	-13.3 to -7.6	0.013
30-49						
RIP	-1.3	-1.8 to -0.8	<0.001	-0.8	-1.3 to -0.3	0.003
%P	-3.2	-5.7 to -0.7	0.015	-4.4	-8.8 to -0.2	0.043
%W	-13.7	-17.9 to -9.6	<0.001	-21.5	-28.2 to -14.5	<0.001
%E	-6	-9.2 to -6	<0.001	-4.5	-9.9 to -4.5	0.095
≥ 50						
RIP	-0.8	-1.1 to -0.4	<0.001	-0.3	-0.5 to 0	0.045
%P	-3.9	-6.2 to -1.7	0.001	-0.2	-3.5 to 3.2	0.909
%W	-9.8	-13.5 to -6.1	<0.001	-12.2	-17.8 to -6.4	<0.001
%E	-7.2	-10.1 to -7.2	<0.001	-8.9	-13.3 to -8.9	<0.001

Supplementary Table 2. Percent changes in current smoking prevalence based on fixed effect coefficients from adjusted mixed effect linear regression models (overall proportion adopted models), by sex and age group.

Models examined the adjusted association between the proportion measures adopted across smoke-free, health warnings, and advertising policy domains (%PWE) and cigarette's affordability (RIP) and current smoking prevalence, from 2009 to 2017, across 175 countries (n=844). Linear mixed models were fit by maximum likelihood and t-tests used Satterthwaite approximations to degrees of freedom. P values were considered statistically significant if lower than 0.05.

	Men			Women		
	Relative change	95% UI	p value	Relative change	95% UI	p value
≥ 15						
RIP	-1.1	-1.5 to -0.7	<0.001	-0.8	-1.2 to -0.4	0.001
%PWE	-20.5	-23.3 to -17.6	<0.001	-25.7	-29.8 to -21.4	<0.001
15-29						
RIP	-1.4	-1.9 to -0.9	<0.001	-1.1	-1.8 to -0.5	0.002
%PWE	-20.5	-23.7 to -17.3	<0.001	-29.5	-34.9 to -23.9	<0.001
30-49						
RIP	-1.3	-1.8 to -0.8	<0.001	-0.8	-1.3 to -0.4	0.002
%PWE	-21.2	-24.1 to -18.1	<0.001	-27.0	-31.7 to -22	<0.001
≥ 50						
RIP	-0.8	-1.1 to -0.4	<0.001	-0.2	-0.5 to 0	0.062
%PWE	-20.3	-22.9 to -17.6	<0.001	-20.0	-23.8 to -16.1	<0.001

Supplementary Table 3. Percent changes in current smoking prevalence based on fixed effect coefficients from adjusted mixed effect linear regression models (compliance models), by policy component, sex and age group. Models examined the adjusted association between smoke-free (P-compliance), direct advertising (E-D-compliance), and indirect advertising (E-I-compliance) compliance scores and cigarette's affordability (RIP) and current smoking prevalence, from 2009 to 2017, across 175 countries (n=305). Linear mixed models were fit by maximum likelihood and t-tests used Satterthwaite approximations to degrees of freedom. P values were considered statistically significant if lower than 0.05.

	Men			Women		
	Relative change	95% UI	p value	Relative change	95% UI	p value
≥ 15						
RIP	-1.0	-1.3 to -0.7	<0.0001	-1.1	-1.6 to -0.6	0.002
P-compliance	-0.2	-0.5 to 0.2	0.380	-0.4	-0.9 to 0.1	0.151
E-D-compliance	-0.1	-0.5 to 0.4	0.752	0.1	-0.4 to 0.7	0.653
E-I-compliance	-0.1	-0.6 to 0.2	0.489	-0.2	-0.8 to 0.4	0.506
15-29						
RIP	-1.0	-1.3 to -0.6	<0·0001	-1.3	-1.8 to -0.7	0.009
P-compliance	-0.2	-0.6 to 0.2	0.378	-0.3	-0.9 to 0.4	0.427
E-D-compliance	0.0	-0.4 to 0.5	0.954	0.3	-0.5 to 1.0	0.442
E-I-compliance	-0.2	-0.6 to 0.2	0.363	-0.4	-1.1 to 0.4	0.293
30-49						
RIP	-1.1	-1.4 to -0.7	<0.0001	-1.5	-2.2 to -0.8	0.002
P-compliance	-0.2	-0.6 to 0.2	0.357	-0.7	-1.2 to -0.1	0.036
E-D-compliance	-0.2	-0.6 to 0.3	0.513	0.1	-0.6 to 0.7	0.838
E-I-compliance	-0.1	-0.6 to 0.3	0.519	0.0	-0.7 to 0.6	0.950
≥ 50						
RIP	-0.9	-1.2 to -0.6	<0.0001	-0.7	-1.2 to -0.3	0.004
P-compliance	-0.1	-0.4 to 0.2	0.541	-0.2	-0.7 to 0.3	0.452
E-D-compliance	0.0	-0.4 to 0.3	0.829	0.1	-0.4 to 0.3	0.614
E-I-compliance	-0.1	-0.4 to 0.3	0.732	-0.2	-0.7 to 0.4	0.585

Supplementary Table 4. Percent changes in current smoking prevalence based on fixed effect coefficients from adjusted mixed effect linear regression models (interaction models), by policy aspects, sex and age group.

Models examined the adjusted association between smoke-free (P), health warnings (W), advertising (E) achievements scores, smoke-free (P-compliance) and direct advertising (E-D-compliance) compliance scores, and cigarette's affordability (RIP) and current smoking prevalence, from 2009 to 2017, across 175 countries (n=332); Interaction terms between smoke-free policies achievement and compliance scores (P*P-compliance) and between direct advertising achievement and compliance scores (E*E-D-compliance) were also added as predictors. Linear mixed models were fit by maximum likelihood and t-tests used Satterthwaite approximations to degrees of freedom. P values were considered statistically significant if lower than 0.05.

	Men			Women		
	Relative change	95% UI	p value	Relative change	95% UI	p value
≥ 15						
RIP	-0.4	-0.7 to -0.2	0.003	-0.6	-1.1 to -0.2	0.056
P	-1.4	-3.4 to 0.5	0.162	-2.0	-5.0 to 0.9	0.161
W	-2.0	-2.7 to -1.2	<0·0001	-3.4	-4.3 to -2.4	<0·0001
E	-0.1	-3.6 to 3.3	0.980	-2.7	-7.3 to 2.1	0.287
P-compliance	0.4	-0.4 to 1.1	0.366	-0.4	-1.6 to 0.6	0.406
E-D-compliance	1.0	-0.1 to 0.6	0.083	0.9	-0.7 to 2.5	0.314
P*P-compliance	-0.1	-0.3 to 0.2	0.630	0.1	-0.2 to 0.5	0.431
E*E-D-compliance	-0.3	-0.7 to 0.1	0.103	-0.2	-0.7 to 0.4	0.513
15-29						
RIP	-0.5	-0.7 to -0.2	0.003	-0.5	-0.8 to -0.1	0.006
P	-1.2	-3.4 to 1.0	0.265	-0.1	-3.6 to 3.4	0.968
W	-2.0	-2.8 to -1.2	<0·0001	-3.9	-5.2 to -2.6	<0·0001
E	-0.3	-4.5 to 3.9	0.891	-3.1	-9.5 to 3.2	0.338
P-compliance	0.4	-0.4 to 1.2	0.380	0.2	-1.1 to 1.6	0.724
E-D-compliance	1.0	-0.3 to 2.4	0.118	1.0	-1.1 to 3.0	0.385
P*P-compliance	-0.1	-0.4 to 0.2	0.595	-0.1	-0.6 to 0.4	0.677
E*E-D-compliance	-0.3	-0.8 to 0.1	0.163	-0.2	-0.9 to 0.5	0.636
30-49						
RIP	-0.4	-0.7 to -0.2	0.006	-0.3	-0.6 to 0.0	0.053
P	-1.7	-3.9 to 0.6	0.128	-1.2	-4.4 to 2.1	0.479
W	-2.2	-3.0 to -1.4	<0·0001	-3.6	-4.8 to -2.4	<0·0001
E	0.0	-3.9 to 3.8	0.984	-5.1	-11.3 to 1.3	0.102
P-compliance	0.3	-0.6 to 1.2	0.486	-0.6	-1.9 to 0.7	0.366
E-D-compliance	1.1	-0.3 to 2.3	0.117	0.4	-1.6 to 2.6	0.677
P*P-compliance	0.0	-0.4 to 0.3	0.749	0.1	-0.3 to 0.5	0.722
E*E-D-compliance	-0.4	-0.8 to 0.1	0.122	0.0	-0.7 to 0.7	0.959
≥ 50						
RIP	-0.4	-0.6 to -0.1	0.006	-0.2	-0.5 to 0.0	0.068
P	-1.3	-3.3 to 0.6	0.189	-2.2	-4.8 to 0.4	0.104
W	-1.7	-2.4 to -1.0	<0·0001	-2.8	-3.8 to -1.8	<0·0001
E	-0.1	-3.6 to 3.4	0.927	-4.4	-9.2 to 0.2	0.075
P-compliance	0.5	-0.3 to 1.2	0.222	-0.5	-1.5 to 0.5	0.339
E-D-compliance	1.0	0.0 to 2.2	0.080	0.0	-1.6 to 1.5	0.964
P*P-compliance	-0.1	-0.3 to 0.1	0.497	0.2	-0.1 to 0.5	0.238
E*E-D-compliance	-0.3	-0.7 to 0.1	0.109	0.1	-0.4 to 0.7	0.655
