# Supplementary material for: The effects of tobacco control policies on global smoking prevalence
Source: Nat Med. 2021 Jan 21;27(2):239–43. doi: 10.1038/s41591-020-01210-8 (PMC7884287; doi:10.1038/s41591-020-01210-8)
Supplement: Supplementary file 2 — Reporting Summary [file 41591_2020_1210_MOESM2_ESM.pdf]

nature research

Corresponding author(s): Emmanuela Gakidou

Last updated by author(s): Dec 7, 2020

Reporting Summary

Nature Research wishes to improve the reproducibility of the work that we publish. This form provides structure for consistency and transparency in reporting. For further information on Nature Research policies, see our Editorial Policies and the Editorial Policy Checklist.

Statistics

For all statistical analyses, confirm that the following items are present in the figure legend, table legend, main text, or Methods section.

n/a	Confirmed	
☐	☒	The exact sample size (*n*) for each experimental group/condition, given as a discrete number and unit of measurement
☐	☒	A statement on whether measurements were taken from distinct samples or whether the same sample was measured repeatedly
☐	☒	The statistical test(s) used AND whether they are one- or two-sided<br>*Only common tests should be described solely by name; describe more complex techniques in the Methods section.*
☐	☒	A description of all covariates tested
☒	☐	A description of any assumptions or corrections, such as tests of normality and adjustment for multiple comparisons
☐	☒	A full description of the statistical parameters including central tendency (e.g. means) or other basic estimates (e.g. regression coefficient) AND variation (e.g. standard deviation) or associated estimates of uncertainty (e.g. confidence intervals)
☐	☒	For null hypothesis testing, the test statistic (e.g. *F*, *t*, *r*) with confidence intervals, effect sizes, degrees of freedom and *P* value noted<br>*Give P values as exact values whenever suitable.*
☒	☐	For Bayesian analysis, information on the choice of priors and Markov chain Monte Carlo settings
☐	☒	For hierarchical and complex designs, identification of the appropriate level for tests and full reporting of outcomes
☒	☐	Estimates of effect sizes (e.g. Cohen's *d*, Pearson's *r*), indicating how they were calculated

Our web collection on statistics for biologists contains articles on many of the points above.

Software and code

Policy information about availability of computer code

Data collection	No primary data collection was carried out for this analysis.
Data analysis	All analyses were executed with RStudio (version 1.1.383) using the lmer function in the R package lme4.

For manuscripts utilizing custom algorithms or software that are central to the research but not yet described in published literature, software must be made available to editors and reviewers. We strongly encourage code deposition in a community repository (e.g. GitHub). See the Nature Research guidelines for submitting code & software for further information.

Data

Policy information about availability of data

All manuscripts must include a data availability statement. This statement should provide the following information, where applicable:

- Accession codes, unique identifiers, or web links for publicly available datasets
- A list of figures that have associated raw data
- A description of any restrictions on data availability

The dataset generated and analyzed during the current study is openly available at http://ghdx.healthdata.org/record/ihme-data/global-tobacco-control-and-smoking-prevalence-scenarios-2017 (DOI: 10.6069/QAZ7-6505). The dataset contains all data necessary to interpret, replicate and build upon the methods or findings reported in the article. Tobacco control policy data that support the findings of this study are released every two years as part of WHO's Global Report on Tobacco Control, which are also directly accessible at https://www.who.int/tobacco/global_report/en/.

Field-specific reporting

Please select the one below that is the best fit for your research. If you are not sure, read the appropriate sections before making your selection.

☒ Life sciences ☐ Behavioural & social sciences ☐ Ecological, evolutionary & environmental sciences

For a reference copy of the document with all sections, see nature.com/documents/nr-reporting-summary-flat.pdf

Life sciences study design

All studies must disclose on these points even when the disclosure is negative.

Sample size	Final sample consisted of 823 observations across 175 countries for each sex (males, females, and both sexes) and age (15 plus, 15-29, 30-49, and 50 plus) specific models. No sample size was calculated because all country-years with available smoking prevalence estimate and national level data on cigarette prices and policy achievement scores were included in the analysis. Specifically for the analysis of counterfactual scenarios, all 155 countries with non-missing policy indicators for both 2008 and 2016 were included, as described in the methods section.
Data exclusions	As described in the methods section of the main text, country-year observations were excluded if any indicator - smoking prevalence, cigarette prices, and policy achievement scores - was missing. Out of the 195 countries with estimated smoking prevalence in the 2017 Global Burden of Disease study, 20 had no tobacco control indicators for any year between 2008 and 2016 and were not included in our models. Additional 20 countries were excluded in our counterfactual analysis due to lack of availability of cigarettes prices and/or policy achievement scores in both 2008 and 2016. The decision to exclude these locations from our entire conterfactual analysis was to keep our pooled estimated reduction in prevalence consistent across alternative scenarios (same number of countries were included for each scenario prediction).
Replication	This is an observational study using multiple years of smoking prevalence estimates (2009-2017) and publicly available national-level tobacco control policy data (2008-2016)
Randomization	This analysis is an observational study and there were no experimental groups.
Blinding	Blinding was not relevant to this study, as it was an observational study using survey data and publicly available tobacco control policy indicators.

Reporting for specific materials, systems and methods

We require information from authors about some types of materials, experimental systems and methods used in many studies. Here, indicate whether each material, system or method listed is relevant to your study. If you are not sure if a list item applies to your research, read the appropriate section before selecting a response.

Materials & experimental systems

n/a	Involved in the study	
☒	☐	Antibodies
☒	☐	Eukaryotic cell lines
☒	☐	Palaeontology and archaeology
☒	☐	Animals and other organisms
☒	☐	Human research participants
☒	☐	Clinical data
☒	☐	Dual use research of concern

Methods

n/a	Involved in the study	
☒	☐	ChIP-seq
☒	☐	Flow cytometry
☒	☐	MRI-based neuroimaging
